# From Flow to Feature Using a Proof-of-Concept Spectral-Driven Machine Learning Approach Using Smart Urinary and Drainage Catheter Systems: Algorithm Development and Validation

**DOI:** 10.2196/80829

**Published:** 2026-05-14

**Authors:** Leonardo Poggi, Anastasia Meckler, Sebastian Künert, Julia Jeske, Ramsi Siaj, Thanusiah Selvamoorthy, Michael Fabian Berger, Felix Nensa, Judith Kohnke, Bernadette Hosters, Jennifer Brendt-Müller, Mario Roser, René Hosch

**Affiliations:** 1 Institute of Diagnostic and Interventional Radiology and Neuroradiology, Essen University Hospital, Hufelandstraße 55, Essen, 45147, Germany, 43 20172377817; 2 Institute for Artificial Intelligence in Medicine (IKIM), Essen University Hospital, Essen, Germany; 3 Department of Pediatric Surgery, Essen University Hospital, Essen, Germany; 4 Department of Nursing Development and Nursing Research, Essen University Hospital, Essen, Germany; 5 Elixion Medical GmbH, Düsseldorf, Germany

**Keywords:** AI in medicine, urine diagnostics, surgical drains, spectroscopy, digital health care, real-time monitoring, early warning systems, artificial intelligence

## Abstract

**Background:**

Current urinary and drainage catheter systems collect fluids for visual inspection or manual sampling, offering limited diagnostic value while being labor-intensive and prone to error. Machine learning (ML) has the potential to automate the analysis of these fluids. However, existing methods rely on complex preprocessing steps, which hinder real-time analysis.

**Objective:**

We aim to develop and evaluate a fully automated, real-time diagnostic approach for smart urinary and drainage catheter systems by leveraging spectral data and ML to differentiate pathological from healthy excreted fluids without the need for manual preprocessing.

**Methods:**

This study proposes a novel, fully automated approach for smart urinary and drainage catheter systems that uses spectra and ML to extract features from excreted fluids, enabling real-time analysis directly. A total of 454 surgical drainage fluid samples (from 181 patients) and 401 urine catheter samples (from 168 patients) were analyzed using smart catheters and drains equipped with compact mini-spectrometer sensors. The collected spectral data were fed into 3 different ML models: a random forest, a partial least squares discriminant analysis regression, and a convolutional neural network (CNN). Each model aimed to extract features and differentiate between pathological and healthy urine and drainage samples based on the various biomarkers available from previously conducted laboratory analyses.

**Results:**

All 3 approaches (random forest, partial least squares discriminant analysis regression, and CNN) achieved promising results, demonstrating the potential of the overall approach. In particular, the CNN models trained on the drainage biomarkers hemoglobin and bilirubin achieved the best results. Matthews correlation coefficient scores of 0.83 and 0.81 were obtained for hemoglobin and bilirubin, respectively, when differentiating between pathological and healthy samples using the extracted spectral features.

**Conclusions:**

This work demonstrates the potential of spectral-driven ML for smart urinary and drainage catheter systems. This approach offers a real-time, noninvasive method for analyzing excreted fluids, paving the way for improved diagnostics and personalized patient care. Further research will explore the optimal ML model for this application.

## Introduction

Urinary and drainage catheters are indispensable medical devices widely used across various clinical settings. The primary usage of such devices is to aid the excretion of biological fluids from hospitalized patients. The collected liquids are periodically monitored by health care professionals to gain insight into the patients’ health status. For example, surgical drains are used to evacuate fluid from the postoperative site, allowing for the monitoring of wound healing progress by tracking the volume and quality of the fluid [1]. In the case of urinary catheters, the different urine biomarkers are critical for identifying diseases and underlying conditions [2,3].

While these devices play a crucial role in patient care, their extensive usage can predispose individuals to a variety of complications. In the case of urinary catheters, common complications include urinary tract infections, bladder stones, and urethral injuries [4-6]. Similarly, the usage of drainage catheters can lead to complications such as catheter blockage, leakage, tissue trauma, and infections at the insertion site [7-9]. These complications can lead to prolonged hospital stays and even systemic infections if not promptly identified and addressed.

As of today, the current approach consists of visual inspection and manual sampling of these fluids at specific, discrete time intervals by medical staff. To obtain a quantitative evaluation of the composition of the collected fluids, separate laboratory analyses must be performed. This implies a significant time delay in detecting potentially severe complications. For these reasons, a reliable automation of such monitoring processes would bring along numerous advantages. In times of severe medical staff shortages, automated and continuous monitoring could not only contribute to improved patient monitoring quality but also, at the same time, reduce the monitoring routine workload.

Research has been conducted in this field. For instance, in the case of catheter-associated urinary tract infections, electronic monitoring systems have been developed to accelerate the detection of such complications [10]. Additionally, to enhance the performance of ordinary urine dipstick tests, a machine learning (ML)–based approach has been proposed [11]. For surgical drain outputs, digital solutions have been implemented for volumetric measurements [12] and for determining the fluid’s color [13]. A more comprehensive solution for surgical drain outputs has been proposed by Roser et al [14] by introducing a so-called SmartDrain (Elixion Medical GmbH) device that performs and analyzes spectral measurements on drainage fluids at the patient’s bedside.

In recent years, artificial intelligence (AI) has garnered significant attention in the context of biomedical research. The application of ML methods to such research questions, coupled with the increasing computational power available, has demonstrated remarkable performance in analyzing vast amounts of biomedical data and extracting meaningful features [15,16].

In the present study, we aim to build upon the approach proposed by Roser et al [14] by implementing an AI-driven early warning system for the detection of pathological markers in urine and drainage samples. Specifically, we analyze spectral data acquired with a compact mini-spectrometer using classification algorithms such as partial least squares discriminant analysis regression (PLS-DA), random forest (RF), and convolutional neural networks (CNN). PLS-DA is an established, robust, and highly interpretable classification method mostly used in chemometrics in combination with high-dimensional spectral data [17,18]. RF is a flexible, nonlinear ensemble-based approach capable of modeling complex interactions while maintaining a relatively high degree of interpretability [19]. On the other hand, CNNs represent a data-driven deep learning strategy that has the potential to capture subtle patterns that may not be detectable by more traditional ML approaches [20,21]. Several studies have demonstrated superior performance when processing spectral data with 2D CNNs as opposed to 1D architectures [22-24]. For this reason, in the present study, we implement a simple CNN model that classifies 3-channel images obtained from the raw spectral data.

Together, the selected ML approaches span a wide range of complexity and interpretability, enabling a systematic evaluation of their effectiveness and suitability for spectral-data-based catheter monitoring. Ultimately, our goal is to revolutionize the management of catheter-related complications, improving patient care and health care efficiency.

## Methods

### Study Design

**Figure 1.**
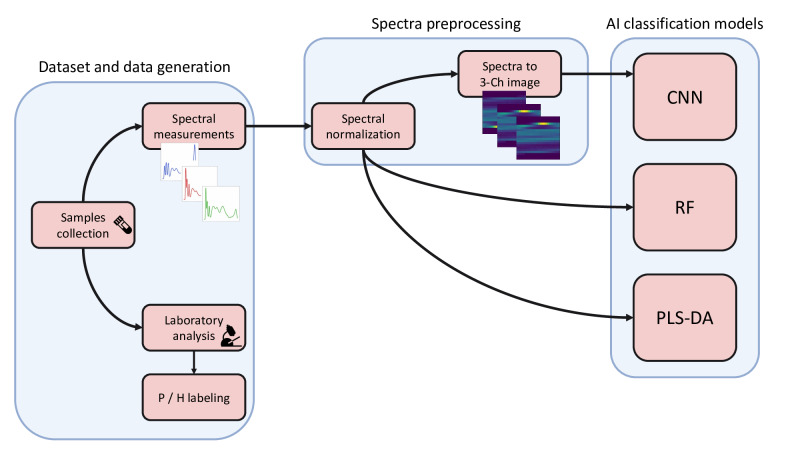
Classification pipeline adopted for this study. In the first step, urine and drainage samples are acquired from patients. For these samples, both spectral measurements and laboratory analysis are performed. To create labeled data, the values of the biomarkers measured in the laboratory are binarized into the categories healthy and pathological. The measured spectra undergo a preprocessing step that includes normalization. The transformed spectra are classified using RF and PLS-DA models. Additionally, the normalized spectra are transformed into 3-channel images, which are then fed to a CNN for classification. Ch: channel; CNN: convolutional neural network; P/H: pathological/health; PLS-DA: partial least squares discriminant analysis regression; RF: random forest.

In Figure 1, the entire pipeline implemented for this study is presented. In the first step, labeled data were generated by acquiring drainage and urine samples and performing spectral measurements as well as laboratory analyses on them. For each collected sample, 3 spectra and a series of laboratory markers were obtained. This labeled data was used to train AI models for each of the laboratory markers (urine and drainage). In the second step of the pipeline, the spectra were preprocessed in preparation for the AI models. This step involved normalizing the spectra. For the CNN model, an additional step was performed by converting the spectra to 3-channel images. The labeled data was eventually fed to 3 separate AI models: a CNN, a PLS-DA, and an RF classification model.

### Ethical Considerations

This study was approved by the Ethics Committee of the University Hospital Essen (21-10402-BO). Informed consent was obtained from all patients. The data included in this study were fully anonymized. Participants received no compensation.

### Dataset and Data Generation

The dataset used in the present work consists of 454 (181 patients) drainage and 401 (168 patients) urine samples. The samples were acquired from patients aged between 0 and 85 years at the University Hospital Essen. The age distribution of the drainage dataset has a median and IQR of 57 and 22, respectively. For the urine data, the median amounts to 56 and the IQR to 20.25.

Each sample was divided into 2 batches. The samples were then frozen and stored at −80°C until further processing. All samples from the first batch were analyzed at the central laboratory of the University Hospital Essen. A total of 14 drainage markers (Table 1) and a total of 11 markers for the urine samples (Table 2) were examined. For each sample, the measured markers were binarized using predefined cutoff values. If a specific marker was higher than the corresponding cutoff value, the marker was labeled as pathologic. Otherwise, it was labeled as healthy. Those cutoff values were defined by the guidelines provided by the central laboratory (version 1.4 dated September 21, 2021). As there are no predefined cutoff values for drainage fluids, standard values for serum were used as a reference for those samples. However, for the markers hemoglobin and erythrocytes, the cutoff value for pathology was set to 0 because their presence in surgical drain fluids is universally considered pathological and may even indicate relevant postoperative bleeding [25].

**Table 1.** Overview of the drainage markers considered in this study. The marker of a sample is categorized as pathological (red) if its value is less than or equal to the corresponding cutoff value. Otherwise, it is marked as healthy (green). Additionally, the ratio between the minority and majority classes of each binarized marker is listed in the table alongside a visual representation of the distribution between pathological (red bars) and healthy samples (green bars).

Drain marker	Cutoff value	Samples, n	Minimum/majority class	Ratios P versus H
Total protein	2.5 g/dL	454	0.99	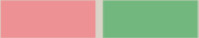
Glucose	50 mg/dL	453	0.72	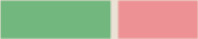
LDH^a^	247 U/L	449	0.54	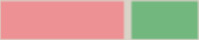
Hemoglobin	0 mg/dL	425	0.5	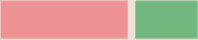
Lipase	53 U/L	454	0.4	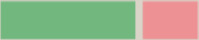
Amylase	53 U/L	447	0.24	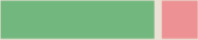
Bilirubin	1.2 mg/dL	453	0.24	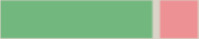
Albumin	2.5 mg/dL	449	0.19	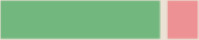
Erythrocytes count	0	427	0.16	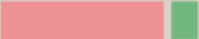
Uric acid	7.2 mg/dL	453	0.09	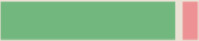
Mononuclear cells	0	420	0.09	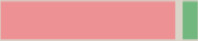
Triglycerides	200 mg/dL	453	0.04	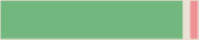
Polymorphonuclear cells	0	420	0.02	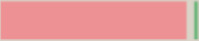
Leucocytes	0/nL	419	0.01	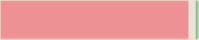

a LDH: lactate dehydrogenase.

**Table 2.** Overview of the urine markers considered in this study. The marker of a sample is categorized as pathological if its value is less than or equal to the corresponding cutoff value. Otherwise, it is marked as healthy. An exception is made for the marker pH, where the healthy samples are found in a range of pH values between 5 and 7.5. Additionally, the ratio between the minority and majority classes of each binarized marker is listed in the table, along with a visual representation of the distribution between pathological (red bars) and healthy samples (green bars).

Urine marker	Cutoff value	Samples, n	Minimum/majority class	Ratios P versus H
Protein	+	401	0.93	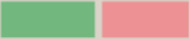
Leucocytes	+	401	0.74	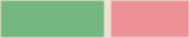
Albumin	2 mg/dL	401	0.32	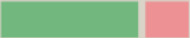
Erythrocytes	+	401	0.24	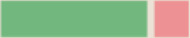
Glucose	16.5 mg/dL	401	0.18	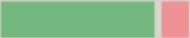
Bilirubin	+	401	0.17	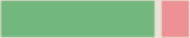
Urobilinogen	0.2 mg/dL	401	0.15	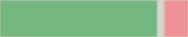
Nitrite	+	401	0.14	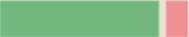
Ketones	+	401	0.13	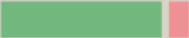
Glucose (stick test)	+	401	0.12	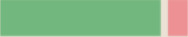
pH	[5, 7.5]	401	0.1	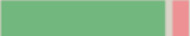

Two exceptions to the binarization procedure were made for the majority of urine markers. Most of these were measured using urine dipstick tests, which estimate the concentration of the marker using a categorical scale. A measurement was marked with the minus symbol “−” if the concentration of the marker is not high enough to be detectable. Pathologic concentrations of the marker were marked with a series of plus symbols “+.” Therefore, a marker was classified as pathologic if the laboratory stick measurement showed at least one “+” symbol. Otherwise, the marker was classified as healthy. The second exception to the binarization procedure was made for pH. This marker does not present a single cutoff value but rather a range of values for which the marker was considered normal and was, therefore, considered healthy. Values measured outside the defined ranges were defined as pathological.

Additionally, in Tables 1 and 2, the ratio between the minority and majority classes of each binarized drainage and urine marker is listed. This value provides important information on the balance between pathological (red bars) and healthy (green bars) samples within a specific fluid marker. Therefore, a perfectly balanced dataset where both classes include the same number of samples would produce a ratio of 1. On the contrary, a dataset where 1 of the 2 classes does not contain any sample would produce a ratio of 0. As shown in the results, this value greatly affects the quality and performance of the trained models.

**Figure 2.**
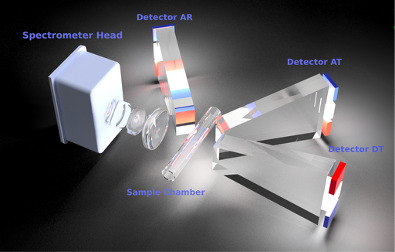
Schematic of the mini spectrometer and its key components. Light from the hyperspectral light sources is directed via the light guides to the sample chamber where it interacts with the sample. The resulting light output is captured by a lens array and focused on the spectrometer head. This setup uses three light sources placed at different angles to form three distinct light paths: direct transmission (DT), angular transmission (AT), and angular reflection (AR). This figure was rendered using Autodesk Fusion 360. Figure rendered with Autodesk Fusion 360 (Autodesk GmbH). AR: angular reflection; AT: angular transmission; DT: direct transmission.

For each sample in the second batch, spectral measurements were performed using a compact mini-spectrometer. The mini-spectrometer was integrated with a self-developed lens array and an electrical current- and temperature-controlled hyperspectral illumination source. This setup ensures broad-spectrum illumination of the samples. The evaluation platform, although relatively large in size, was designed to accommodate the assessment of multiple illumination angles simultaneously and did not yet focus on size reduction during its development. A schematic representation of the spectrometer is presented in Figure 2.

**Figure 3.**
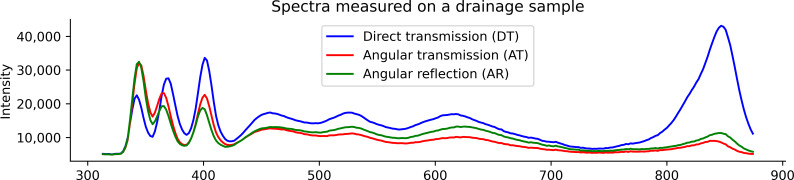
Example spectral measurement on a drainage sample. Each sample is illuminated from 3 different angles, obtaining 3 spectra: DT (blue curve), AT (red curve), and AR (green curve). AR: angular reflection; AT: angular transmission; DT: direct transmission.

The measured spectra consist of 288 data points captured between 313.08 and 874.27 nm. The data collection was improved by illuminating each sample from 3 different angles: direct transmission (DT), angular transmission (AT), and angular reflection (AR). The exposure time for each angle was fine-tuned to achieve an optimal signal-to-noise ratio, amounting to 20, 200, and 320 Âµs for the settings DT, AT, and AR, respectively. Therefore, the input data for the AI models can be thought of as a feature matrix with the shape of (N, 3, 288). Where N is the number of samples of a specific fluid marker, 3 is the number of measured spectra per sample (DT, AT, and AR), and 288 is the number of datapoints measured for each spectrum. An example measurement of a drainage sample is shown in Figure 3.

Following each measurement, the sample was discarded, and the evaluation platform’s tubing was thoroughly flushed with sterile water to prevent cross-contamination between samples. After completing the batch measurement, the tubing was flushed with isopropyl alcohol followed by water to prevent sample cross-contamination and bacterial growth.

### Spectra Preprocessing

#### Spectra Normalization

**Figure 4.**
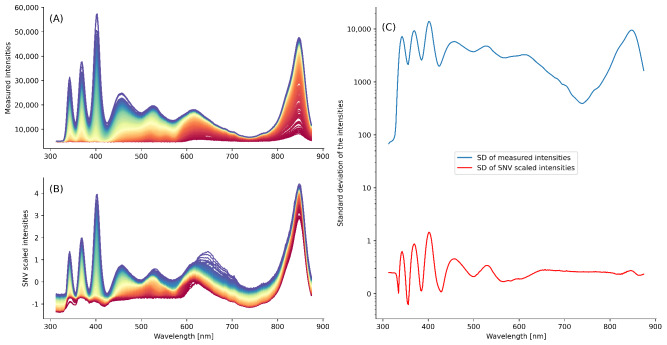
SNV correction applied to the spectral data. (A) Original spectra. (B) Spectra after the SNV correction. (C) Semilogarithmic plot of the SD of the intensities before (blue line) and after (red line) the SNV correction. SNV: standard normal variate;

In the present work, the effect of bias in the input data was mitigated by scaling the spectra using the standard normal variate (SNV) method [26]. Therefore, all spectra were transformed into new spectra with 0 mean and unit variance, as shown in Figure 4A-C. For each wavelength, the SD of the intensities of the spectra before (blue line) and after (red line) the SNV correction is shown in a semilogarithmic plot. For all spectrometer settings (DT, AT, and AR) and liquid type, a drastic reduction in the SD of the intensities is obtained, from approximately 2500 (before SNV) to 0.2 (after SNV).

For each spectrometer setting, the drainage spectra exhibited a higher spectral variability when compared to the urine spectra. Quantitatively, the mean of the wavelength-wise intensity SDs showed increases of approximately 53% (DT), 140% (AT), and 133% (AR) for drainage relative to urine samples. A visual representation of this behavior is presented in Figure S1 in Multimedia Appendix 1.

#### Spectra to Image Conversion

**Figure 5.**
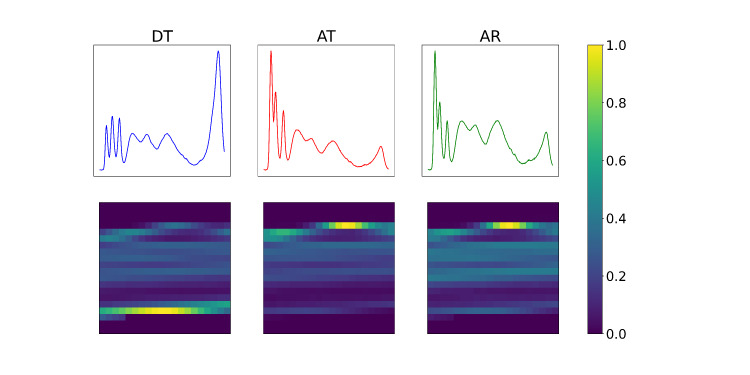
Spectra to image conversion. The 3 spectra measured on each sample are transformed into a 3-channel image by reshaping and padding the arrays of intensity values. Those images were used as input for the CNN architecture. AR: angular reflection; AT: angular transmission; CNN: convolutional neural network; DT: direct transmission.

For the CNN classification models, 3-channel images from the measured spectra were generated. The 3 spectra of each sample can be thought of as a 2D array with the shape of (3, 288). After normalizing the spectra in the range [0, 1], symmetric 0 padding with a length of 56 was applied to the second dimension of the array to reach a total length of 400. After that, the array is reshaped into the shape (3, 20, 20; Figure 5).

### AI Classification Models

For each fluid marker dataset, the samples were grouped at the patient level to ensure that all measurements from a given patient were kept together. Thereafter, a patient-wise train-test split with an 80/20 ratio was performed. This approach prevents data leakage, ensuring that all samples measured on a specific patient are not spread across multiple datasets [27,28]. Consequently, the training set was further subdivided into 3 nonoverlapping subsets, grouped at the patient level and stratified with respect to the target label (eg, ratio between pathological and healthy subjects). Those datasets were used for cross-validation training. Each fold was then tested on the test set, and the classification probabilities were averaged across all 3 folds to obtain the final classification probabilities on the test data.

Due to the highly imbalanced datasets of many fluid markers, the Matthews correlation coefficient (MCC) was chosen as the primary metric to track and maximize. Alongside MCC, the *F*_1_-score and the true positive rate (TPR) were measured. Additionally, the receiving operating characteristic (ROC) curve was computed together with the corresponding area under the curve (ROCAUC) of each model. Additionally, the predictability of the trained models was assessed by comparing them to a dummy classifier.

### Models

#### About PLS-DA

The partial least square (PLS) algorithm is a regression-based method widely used in near-infrared spectroscopy and chemometrics [17]. Unlike principal component regression, PLS presents the advantage of maximizing the covariance between the transformed input features and the target labels. If the target label is categorical, we speak of PLS-DA [18]. In this study, PLS-DA models for classification were implemented using the following procedures. First, the feature matrix with a shape of (N, 3, 288) was transformed into a 2D matrix with a shape of (N, 864) by concatenating the 3 spectra across the wavelength dimension. In the second step, the whole training set was used to compute up to 50 PLS-DA models. In each iteration, the number of PLS components was increased by one, and the MCC score was measured. Therefore, by maximizing the MCC score, the optimal number of PLS components for a specific model was found. In the final step, a PLS-DA model trained with the optimal number of components identified previously was tested on the test set using 3-fold cross-validation. Additionally, the PLS coefficients of the models were examined to enhance explainability and determine which wavelengths are most crucial for the models’ classifications. All PLS-DA models were implemented using the Python (Python Software Foundation) library Scikit-learn (version 1.3.1).

#### About RF

RF is a popular ML algorithm based on decision trees. With appropriate data, it can deliver robust, highly explainable models, and it is fast to train without needing much computational power [19]. In the present study, RF models were trained in 3-fold cross-validation on N X 864 feature matrices of the different fluid markers. Where N is the number of samples, and 864 are the intensity values of the 3 spectra measured on the samples. Together with the metrics mentioned in the evaluation strategy, the models’ feature importances were tracked to understand which wavelengths contribute the most to the classification’s decision. The implementation of the RF models has been conducted using the Python library Scikit-learn (version 1.3.1). The chosen hyperparameters are listed in Table S3 in Multimedia Appendix 1.

#### About CNN

The third ML approach involves the training of a simple CNN. The architecture consisted of 2 2D convolutional layers and 2 fully connected layers. After each convolution operation, batch normalization and max pooling operations were performed. The 3-channel images obtained from the spectra (see the section About RF) were used as input for the CNN. To obtain a classification probability, the output of the fully connected layer was normalized to a value between 0 and 1 using the sigmoid function. The hyperparameters of the CNN (learning rate, batch size, and optimizer) were determined by means of a manual search guided by the mean MCC computed across all biomarker models trained and evaluated on the whole training set. Using this single set of hyperparameters, separate CNN models were trained for each fluid marker in 3-fold cross-validation, with the model’s weights randomly reinitialized at the beginning of each fold. All CNN models were implemented using the Python library PyTorch (version 1.13.1). An overview of the implemented architecture, along with the training parameters, is provided in Table S4 in Multimedia Appendix 1.

## Results

### Overview

In Table 3, an overview of the performance of the AI models trained on all drainage markers is presented. For each of the markers, hemoglobin, bilirubin, albumin, lactate dehydrogenase, total protein, and mononuclear cells, at least 1 model was trained for which the MCC score measured on the test dataset was higher than 0.5. For those markers, *F*_1_-scores of 0.71 or higher were measured for at least 1 of the AI models. Except for mononuclear cell models, the ROCAUCs of the mentioned markers were consistently higher than 0.8 on all 3 AI approaches. To assess the overall performance of the 3 AI approaches, the average value of the MCC scores across all fluid markers was computed. The PLS-DA and RF methods yield comparable mean MCC scores of 0.37 (SD 0.28) and 0.37 (SD 0.26), respectively, whereas the CNN approach scores slightly higher at 0.4 (SD 0.3). A Friedman test showed no statistically significant difference in performance between the models (*χ*²_2_=2.31, *P*=.315, Kendall *W*=0.08).

**Table 3.** Overview of the results of the PLS-DA^a^, RF^b^, and CNN^c^ models trained and tested on the drainage markers datasets.

	MCC^d^			*F*_1_-score			ROCAUC^e^			TPR^f^		
Drainage marker	PLS-DA	RF	CNN	PLS-DA	RF	CNN	PLS-DA	RF	CNN	PLS-DA	RF	CNN
Hemoglobin	0.78	0.78	0.83	0.95	0.95	0.96	0.97	0.95	0.97	0.96	0.98	0.99
Bilirubin	0.81	0.75	0.81	0.86	0.79	0.85	0.94	0.96	0.95	0.83	0.65	0.74
Albumin	0.29	0.48	0.71	0.41	0.56	0.71	0.75	0.87	0.88	0.55	0.64	0.55
LDH^g^	0.47	0.7	0.65	0.81	0.91	0.88	0.79	0.91	0.89	0.79	0.94	0.85
Total protein	0.61	0.48	0.58	0.78	0.71	0.75	0.85	0.8	0.85	0.81	0.74	0.71
Mononuclear cells	0.46	0.42	0.51	0.96	0.95	0.96	0.63	0.57	0.76	0.97	0.96	0.98
Triglycerides	0.7	0.49	0.49	0.67	0.5	0.5	0.97	0.98	0.97	0.5	0.5	0.5
Lipase	0.3	0.19	0.36	0.56	0.51	0.42	0.71	0.66	0.62	0.48	0.45	0.27
Glucose	0.17	0.29	0.35	0.55	0.55	0.6	0.51	0.69	0.69	0.56	0.47	0.53
Erythrocytes count	0.14	0.1	0.22	0.93	0.94	0.95	0.77	0.89	0.81	0.92	0.96	0.96
Amylase	0.12	0.14	0.08	0.27	0.28	0.08	0.39	0.45	0.48	0.21	0.21	0.04
Uric acid	0	0	0	0	0	0	0.57	0.56	0.61	0	0	0
Polymorphonuclear cells	0	0	0	0.99	0.99	0.99	0.15	1	1	1	1	1
Leukocytes	0.34	0.4	0	0.96	0.97	0.99	1	0.98	0.95	0.93	0.95	1

a PLS-DA: partial least squares discriminant analysis regression.

b RF: random forest.

c CNN: convolutional neural network.

d MCC: Matthew correlation coefficient.

e ROCAUC: area under the curve.

f TPR: true positive rate.

g LDH: lactate dehydrogenase.

The models trained on hemoglobin and bilirubin data stand out from the remaining markers, showing the highest performance measured in this study. The CNN approach performed best for hemoglobin data with an MCC score of 0.83, *F*_1_-score of 0.96, ROCAUC of 0.97, and a TPR of 0.99. The CNN approach delivered promising results also on bilirubin data: MCC score of 0.81, *F*_1_-score of 0.85, ROCAUC of 0.95, and a TPR of 0.74.

**Figure 6.**
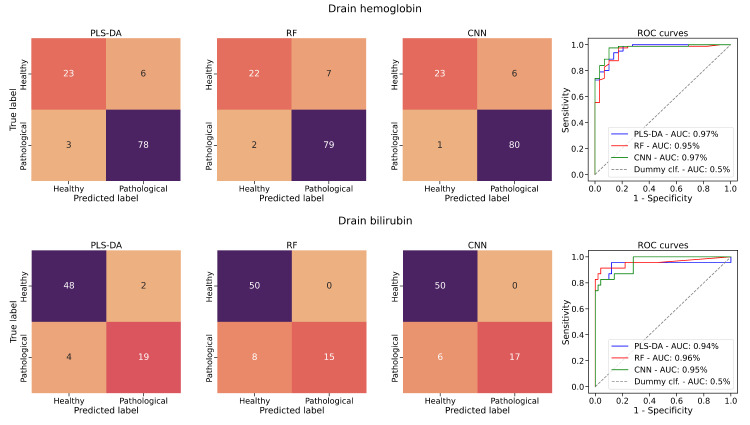
Confusion matrices and ROC curves. Confusion matrices and ROC curves of the PLS-DA, RF, and CNN models trained on drainage hemoglobin and bilirubin data. AUC: area under the curve; CNN: convolutional neural network; PLS-DA: partial least squares discriminant analysis regression; RF: random forest; ROC: receiver operating characteristic.

In Figure 6, the confusion matrices for drainage bilirubin and hemoglobin classification on the test dataset are shown, along with the ROC curves calculated from the predicted classification probabilities. The confusion matrices and ROC curves of the other drainage and urine marker models can be inspected in Figures S2 and S3 in Multimedia Appendix 1.

The models trained on urine data exhibit overall lower performance when evaluated on the test datasets. The mean MCC scores measured across all markers amount to 0.29 (SD 0.26; PLS-DA), 0.28 (SD 0.29; RF), and 0.31 (SD 0.27; CNN). A Friedman test showed no statistically significant difference in performance between the models, with a small to moderate effect size (*χ*²_2_=5.15, *P*=.08, Kendall *W*=0.23). Nevertheless, the markers bilirubin, erythrocytes (hemoglobin), protein, urobilinogen, and albumin were able to achieve MCC scores higher than 0.5. The highest MCC score recorded is achieved with the RF model on bilirubin data, reaching a value of 0.74. An overview of the urine models' performances is presented in Table 4. Overall, the models trained on urine data show clearly less predictive power than the drainage models.

**Table 4.** Overview of the results of the PLS-DA^a^, RF^b^, and CNN^c^ models trained and tested on the urine markers datasets.

	MCC^d^			*F*_1_-score			ROCAUC^e^			TPR^f^		
Urine marker	PLS-DA	RF	CNN	PLS-DA	RF	CNN	PLS-DA	RF	CNN	PLS-DA	RF	CNN
Bilirubin	0.57	0.74	0.63	0.57	0.77	0.67	0.98	0.98	0.98	0.43	0.71	0.57
Erythrocytes or hemoglobin	0.51	0.51	0.61	0.52	0.52	0.59	0.76	0.8	0.95	0.37	0.37	0.42
Protein	0.46	0.46	0.54	0.71	0.72	0.74	0.81	0.8	0.82	0.7	0.74	0.7
Urobilinogen	0.63	0.45	0.53	0.67	0.47	0.56	0.95	0.84	0.84	0.64	0.36	0.45
Albumin	0.38	0.43	0.5	0.46	0.53	0.58	0.75	0.78	0.77	0.35	0.43	0.48
Leucocytes	0.3	0.36	0.4	0.42	0.59	0.44	0.66	0.7	0.72	0.29	0.55	0.29
Nitrite	0.34	0.34	0.25	0.24	0.24	0.22	0.74	0.56	0.88	0.13	0.13	0.13
Glucose	0.23	−0.12	0	0.27	0	0	0.57	0.47	0.25	0.2	0	0
Glucose (stick test)	−0.09	−0.07	0	0	0	0	0.42	0.39	0.43	0	0	0
Ketones	−0.08	−0.04	0	0	0	0	0.52	0.13	0.24	0	0	0
pH	−0.04	0	0	0	0	0	0.72	0.75	0.81	0	0	0

a PLS-DA: partial least squares discriminant analysis regression.

b RF: random forest.

c CNN: convolutional neural network.

d MCC: Matthew correlation coefficient.

e ROCAUC: area under the curve.

f TPR: true positive rate.

### PLS-DA Coefficients and RF Feature Importances

**Figure 7.**
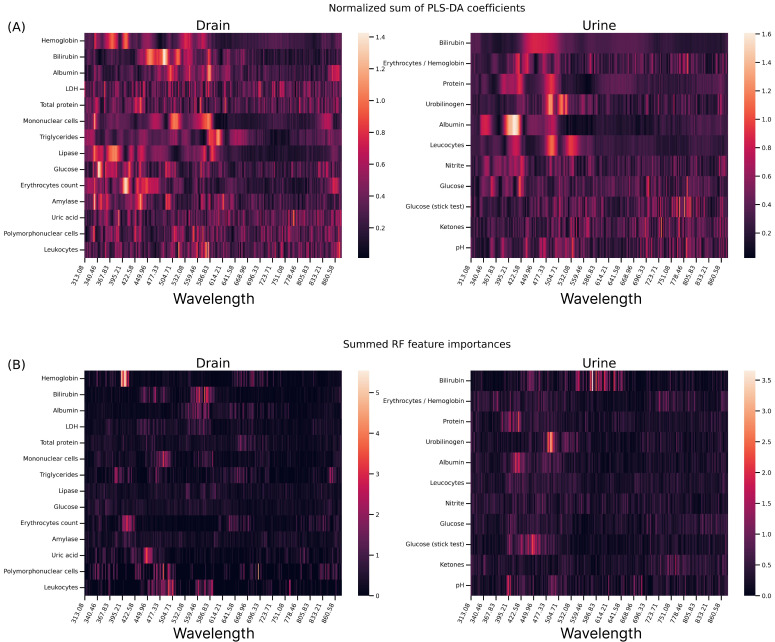
PLS-DA coefficients and RF feature importances. (A) Heatmap representation of the absolute value of the PLS-DA regression coefficients of each drainage and urine model. (B) Heatmap representation of the summed feature importances of each drainage and urine RF model. LDH: lactate dehydrogenase; PLS-DA: partial least squares discriminant analysis regression; RF: random forest.

To improve explainability, the absolute values of the regression coefficients in the PLS-DA models were examined. For each fluid marker model, the values of each PLS coefficient were normalized to the sum of all of the 288 (number of wavelengths) × 3 (light pathways DT, AT, and AR) coefficients. Afterward, for each wavelength, the contributions of the coefficients for the different lightway paths (DT, AT, and AR) were summed to obtain a single value for each wavelength and fluid marker. In Figure 7A, the percentage contribution of the normalized sum of the PLS-DA coefficients is displayed in the form of a heatmap.

In Figure 7B, the RF feature importance values for each fluid marker model are examined. Similarly to the PLS-DA coefficients, the feature importances of the 3 lightway paths (DT, AT, and AR) were summed wavelength-wise. As the sum of all feature importances of the trained models is already equal to 1, there was no need to further normalize the contribution of each wavelength. The summed coefficients were then arranged into a heatmap.

A one-to-one comparison of the PLS-DA and RF heatmaps is not advisable because the PLS coefficients and the RF feature importances represent different statistical concepts (regression vs decision trees). Nevertheless, similar responses are visible when examining the RF feature importances and PLS-DA coefficients of the studied markers. For instance, the summed RF feature importances of drainage bilirubin exhibit 2 distinct peaks, located in the proximity of 470 and 570 nm. These peaks are also clearly visible for the RF model trained on urine bilirubin data. To some extent, similar responses can be seen in the PLS-DA coefficients of the same marker. Another noticeable example is given by hemoglobin. The bright peak at approximately 400 nm, visible in the summed RF feature importances of drainage hemoglobin, can also be identified in the PLS-DA coefficients of the marker. Interestingly, the same peak is clearly visible for the marker erythrocyte count on drainage data. Most probably, this is a consequence of the correlation between the number of blood cells in the samples and the total amount of hemoglobin [29,30].

## Discussion

In the present work, an ML-based approach for classifying urine and drainage samples is presented. Using a compact mini-spectrometer that integrates into ordinary catheter tubes, spectral data were acquired from the samples and used as input for training ML models. This approach plays a key role in the development of an early warning system that could enable real-time monitoring of the composition of the collected liquids.

The results of this study demonstrate that the adopted methodology has significant potential for addressing the investigated research question. Promising AI models were trained for several fluid marker datasets. In particular, MCC scores of at least 0.5 were measured for at least 6 of the 14 drainage markers and 5 of the 11 urine markers. Those models show clear predictive power when classifying the unseen test data. Therefore, it can be confidently stated that the inspected data contains pivotal information for the classification process. Especially relevant are the results obtained on hemoglobin and bilirubin data. Those fluid markers stand out from the rest, showing excellent performance with all 3 AI approaches.

Overall, all 3 approaches led to comparable performances, with the CNN method producing slightly higher scores. As shown in Figure 7, the PLS-DA and RF algorithms offer the benefit of being highly explainable, as they consider the regression coefficients (PLS-DA) and feature importances (RF), respectively. The same wavelength regions seem to play an important role in the model’s decision for many of the markers, but in the case of the PLS-DA models, the responses are more widespread across multiple wavelengths. Put differently, the RF models are, in general, more selective than the PLS-DA ones. The implemented CNN architecture is relatively shallow and small in size. This offers the great advantage of being highly portable and can therefore be easily embedded in microcontrollers for a smart catheter device.

This study has some limitations. In general, the models trained with drainage data as input exhibited better performance than those trained on urine data. This discrepancy in performance is most probably the consequence of the higher spectral variability shown by the drainage spectra (see also Figure S1 in Multimedia Appendix 1) and could be mitigated by experimenting with liquid-specific spectrometer exposure times. Furthermore, as shown in Tables 1 and 2, the distribution between healthy and pathological samples is heavily imbalanced for many fluid markers. For this reason, the trained models for fluid markers, where the imbalance is particularly pronounced, exhibit unsatisfactory performance when tested on the test data. Additionally, the performance of the models is influenced by the adopted dataset-splitting strategy. As described previously, a grouped train-test split and cross-validation are performed based on patient information to prevent data leakage. However, this process further worsens the ratio between the minority and majority label classes of many of the fluid markers datasets, leading to poorly representative training and testing datasets. For more information, consult the Tables S1 and S2 in Multimedia Appendix 1.

For these reasons, the adoption of the MCC score is crucial for accurately interpreting the model’s performance. On the other hand, the *F*_1_-score, the ROCAUC, and the TPR offer only a limited view on the real performances of the trained models and prove to be reliable metrics only on the datasets that show less pronounced imbalances. Models with high MCC scores tend to exhibit high ROCAUCs, *F*_1_-scores, and TPRs. However, the opposite is not always true. The ROC curve, the *F*_1_-score, and the TPR are biased toward positive samples (ie, pathological samples) [31-33]. Therefore, models trained on heavily imbalanced datasets with low MCC scores will tend to show high ROCAUCs, *F*_1_-scores, and TPRs if the majority class of the dataset is represented by pathological samples. For those reasons, the MCC score is the most trustworthy of the considered performance metrics and should be prioritized, especially when examining the results of models trained on highly imbalanced datasets.

Further work is needed to solidify and confirm the findings presented in this work. The datasets should be improved by increasing the number of samples with the aim of reducing the imbalance between pathological and healthy samples. Additionally, increasing the total number of samples would be beneficial to ensure the generation of more representative training, validation, and test datasets. While the current results are promising, further experimentation with several elements of the classification pipeline may help to boost performance. In the first place, different spectral preprocessing techniques could be tested with the aim of enhancing spectral quality [34,35]. Furthermore, the 3 AI algorithms could be further fine-tuned. In particular, a variable selection method could be applied to the PLS-DA algorithm. This method iteratively removes from the input feature matrix the intensity values of the wavelengths that contribute less to the classification’s decision, to optimize the MCC score. Both RF and the CNN method would benefit from standard regularization techniques to reduce overfitting in the validation phase [36]. These refinements could lead to higher performances, especially in fluid markers, where the balance between positive and negative samples is improved by the inclusion of new samples. Another architectural design change worth exploring would be the implementation of a multitask classification framework with the intent of simultaneously modeling multiple biomarkers by learning features from the same spectral input. However, at the present stage, given the limitations regarding sample size and class imbalances, the current, more conservative approach of training a separate classifier for each biomarker is more robust and offers greater interpretability.

Concluding, we value our work as a highly successful proof-of-concept with significant clinical implications. This study represents an important initial step in the development of an early warning system integrated into a smart catheter device. The successful implementation of such a vision has the potential to drastically improve everyday clinical practice by reducing the workload of health care professionals and offering an early detection of urinary and drainage-related complications.

## Supplementary material

10.2196/80829 Multimedia Appendix 1 Additional tables and figures.

## Abbreviations

AI: artificial intelligence
AR: angular reflection
AT: angular transmission
CNN: convolutional neural network
DT: direct transmission
MCC: Matthew correlation coefficient
ML: machine learning
PLS: partial least square
PLS-DA: partial least squares discriminant analysis regression
RF: random forest
ROC: receiving operating characteristic
ROCAUC: area under the curve
SNV: standard normal variate
TPR: true positive rate
