# Supplementary material for: From Flow to Feature Using a Proof-of-Concept Spectral-Driven Machine Learning Approach Using Smart Urinary and Drainage Catheter Systems: Algorithm Development and Validation
Source: JMIR Med Inform. 2026 May 14;14:e80829. doi: 10.2196/80829 (PMC13175236; doi:10.2196/80829)
Supplement: Multimedia Appendix 1 — Additional tables and figures. [file medinform-v14-e80829-s001.docx]

## **Table S1.** Distribution between pathological and healthy drainage samples before and after grouped data splitting on patient basis.

|  | **min. / maj. class** | | |
| --- | --- | --- | --- |
| **Drain Marker** | **Whole Dataset** | **Training Dataset** | **Test Dataset** |
| **Total protein** | 0.99 | 0.93 | 0.74 |
| **Glucose** | 0.72 | 0.7 | 0.78 |
| **LDH** | 0.54 | 0.56 | 0.5 |
| **Hemoglobin** | 0.5 | 0.56 | 0.36 |
| **Lipase** | 0.4 | 0.34 | 0.82 |
| **Amylase** | 0.24 | 0.22 | 0.34 |
| **Bilirubin** | 0.24 | 0.21 | 0.46 |
| **Albumin** | 0.19 | 0.2 | 0.18 |
| **Erythrocytes count** | 0.16 | 0.19 | 0.09 |
| **Uric acid** | 0.09 | 0.08 | 0.18 |
| **Mononuclear cells** | 0.09 | 0.08 | 0.1 |
| **Polymorphonuclear cells** | 0.02 | 0.02 | 0.01 |
| **Triglycerides** | 0.04 | 0.04 | 0.03 |
| **Leukocytes** | 0.01 | 0.04 | 0.03 |

**Table S1.** Ratio between the number of samples in the minority and the majority class of the different drainage markers datasets before (whole dataset) and after (training and test datasets) the data splitting. The patient-based splitting strategy prevents data leakage by grouping all samples measured on the same patient in the same dataset. However, this procedure often worsens the distribution between the pathological and healthy samples.

## **Table S2.** Distribution between pathological and healthy urine samples before and after grouped data splitting on patient basis.

|  | **min. / maj. class** | | |
| --- | --- | --- | --- |
| **Urine Marker** | **Whole Dataset** | **Training Dataset** | **Test Dataset** |
| **Protein** | 0.93 | 0.93 | 0.92 |
| **Leucocytes** | 0.74 | 0.77 | 0.66 |
| **Albumin** | 0.32 | 0.32 | 0.32 |
| **Erythrocytes / Hemoglobin** | 0.24 | 0.24 | 0.25 |
| **Glucose** | 0.18 | 0.2 | 0.32 |
| **Bilirubin** | 0.17 | 0.17 | 0.17 |
| **Urobilinogen** | 0.15 | 0.16 | 0.13 |
| **Nitrite** | 0.14 | 0.14 | 0.19 |
| **Ketones** | 0.13 | 0.15 | 0.07 |
| **Glucose (stick test)** | 0.12 | 0.15 | 0.04 |
| **pH** | 0.1 | 0.08 | 0.16 |

**Table S2.** Ratio between the number of samples in the minority and the majority class of the different urine markers datasets before (whole dataset) and after (training and test datasets) the data splitting. The patient-based splitting strategy prevents data leakage by grouping all samples measured on the same patient in the same dataset. However, this procedure often worsens the distribution between the pathological and healthy samples.

## **Table S3.** RF model parameters.

| **RF Parameter** | **Settings** |
| --- | --- |
| Nr. Estimators | 100 |
| Criterion | Gini |
| Max Depth | None |
| Max Features | Sqrt |
| Min Samples Split | 2 |
| Min Samples Leaf | 1 |
| Bootstrap | True |

**Supplementary Table 3:** Random Forest parameters.

## **Table S4.** CNN model parameters and architecture.

| **CNN Parameter** | **Settings** | |
| --- | --- | --- |
| Image Size | (3, 20, 20) | |
| Learning Rate | 0.0001 | |
| Batch Size | 2 | |
| Epochs | 500 | |
| Optimizer | Stochastic Gradient Descent (SGD) | |
| **CNN Layer** | **Settings** | **Activation Function** |
| Conv2D | in=3, out=16, kernel=3, stride=1, padding=1 | Rectifier Linear unit (ReLu) |
| BatchNorm2D | nr_features=16 |  |
| MaxPool2D | kernel=2, stride=2 |  |
| Conv2D | in=16, out=32, kernel=3, stride=1, padding=1 | Rectifier Linear unit (ReLu) |
| BatchNorm2D | nr_features=32 |  |
| MaxPool2D | kernel=2, stride=2 |  |
| Linear | in=800, out=64 | Rectifier Linear unit (ReLu) |
| Linear | in=64, 1 | Sigmoid |

**Table S4.** CNN architecture and chosen training parameters.

## **Figure S1.** Spectral variability of the normalized drainage and urine spectra.


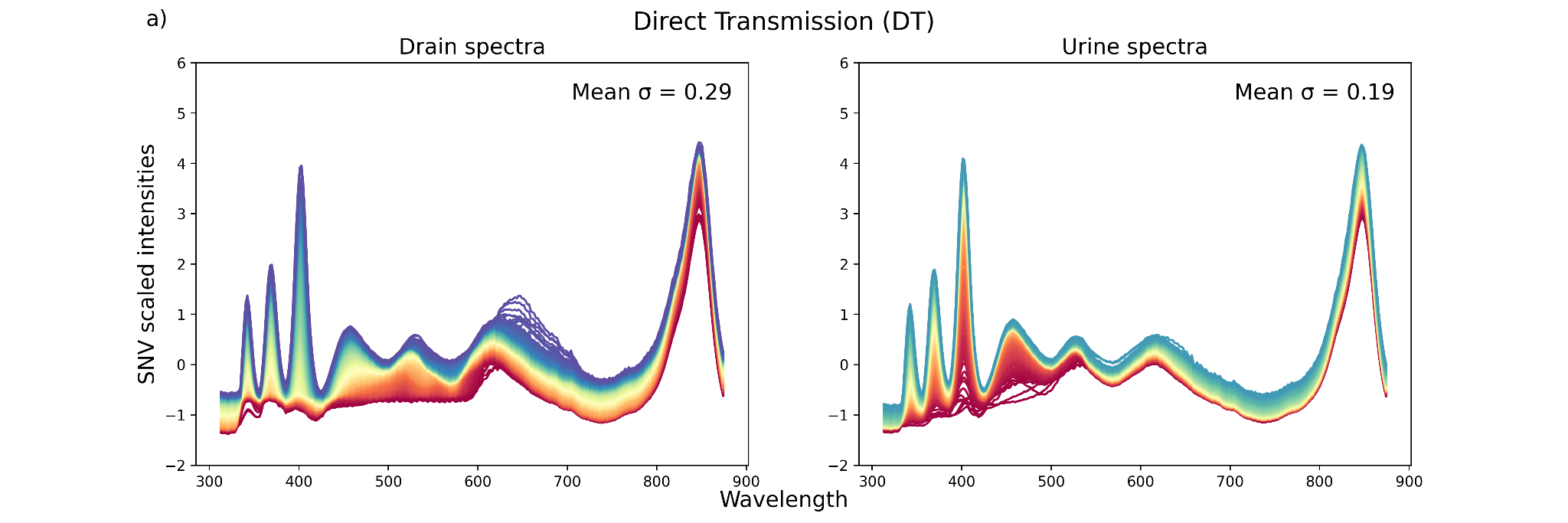


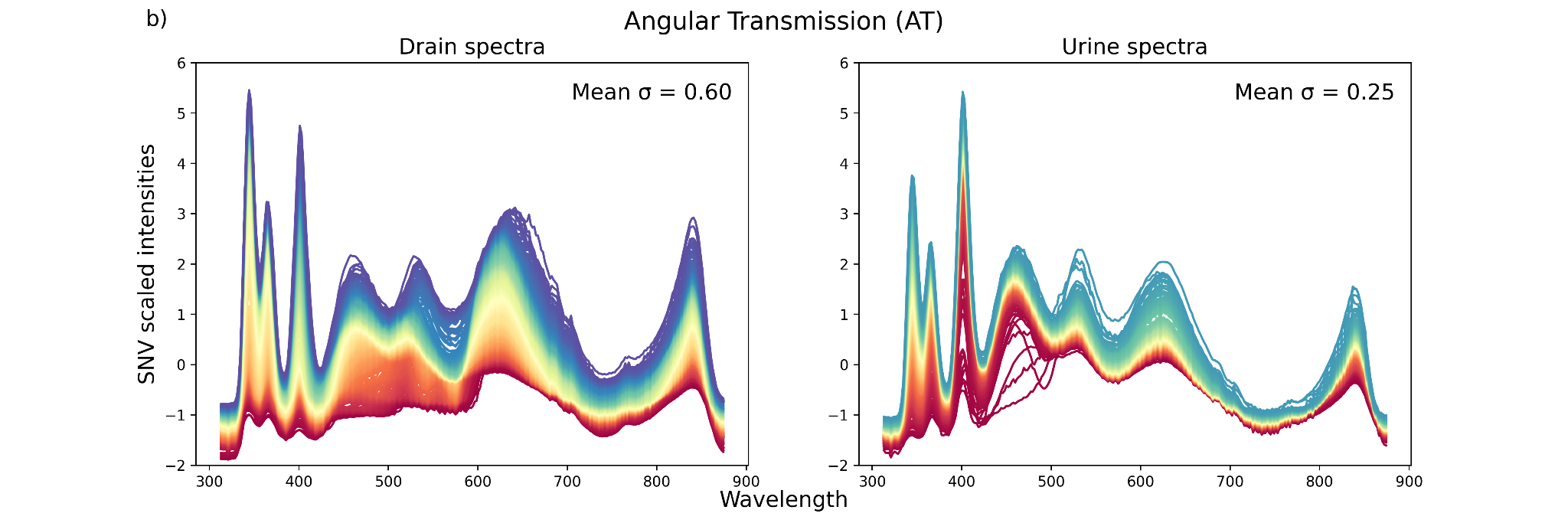


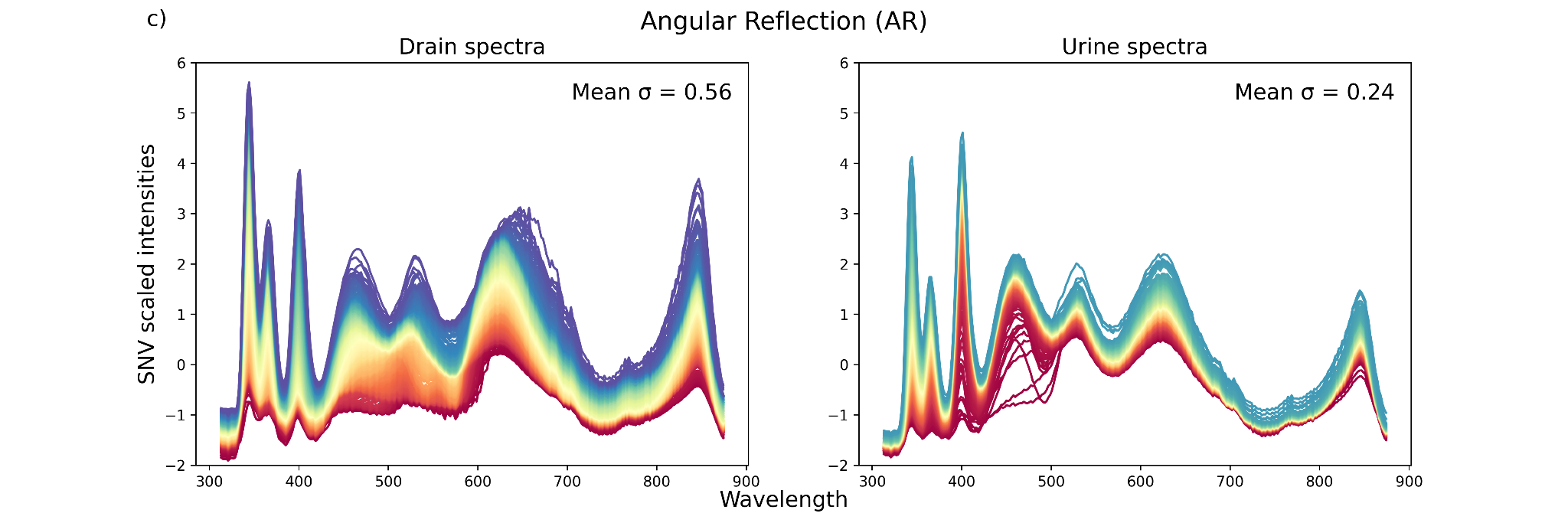


**Figure S1.** Comparison of normalized drainage and urine spectra for the different spectrometer settings. Drainage spectra consistently show higher spectral variability than urine spectra, as quantified by the mean value of the wavelength-wise standard deviations of the intensities (σ).

## **Figure S2.** Confusion matrices and ROC curves of drainage biomarkers models.


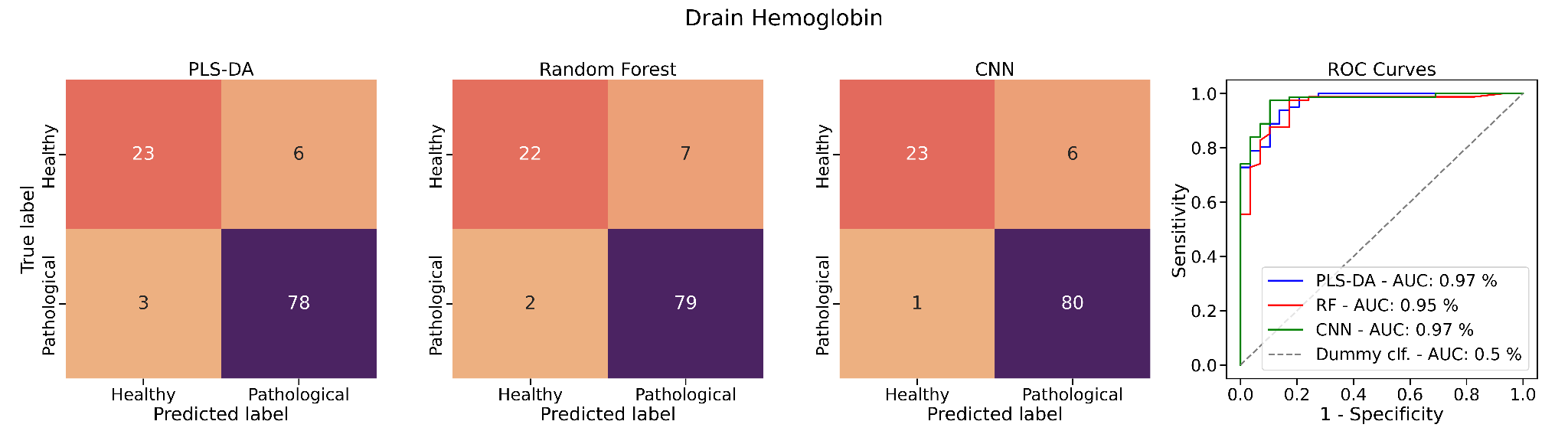

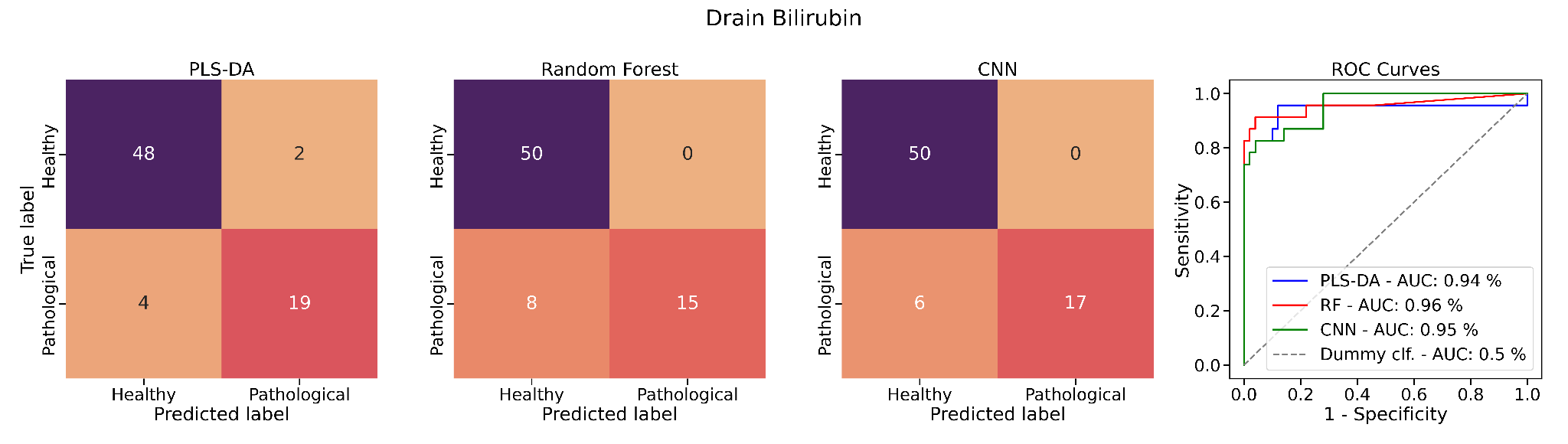

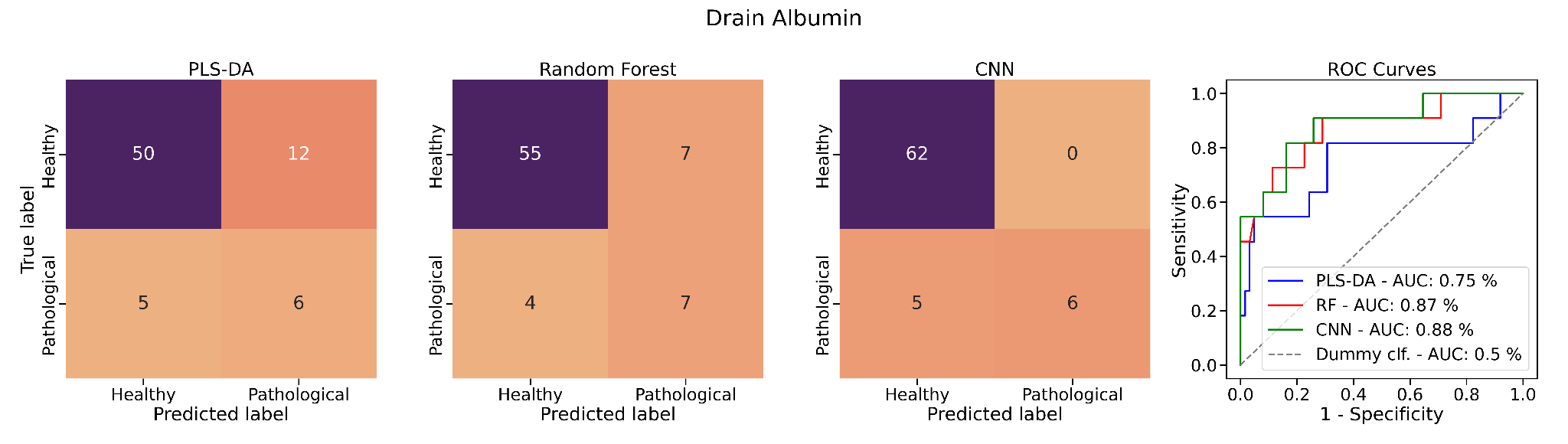


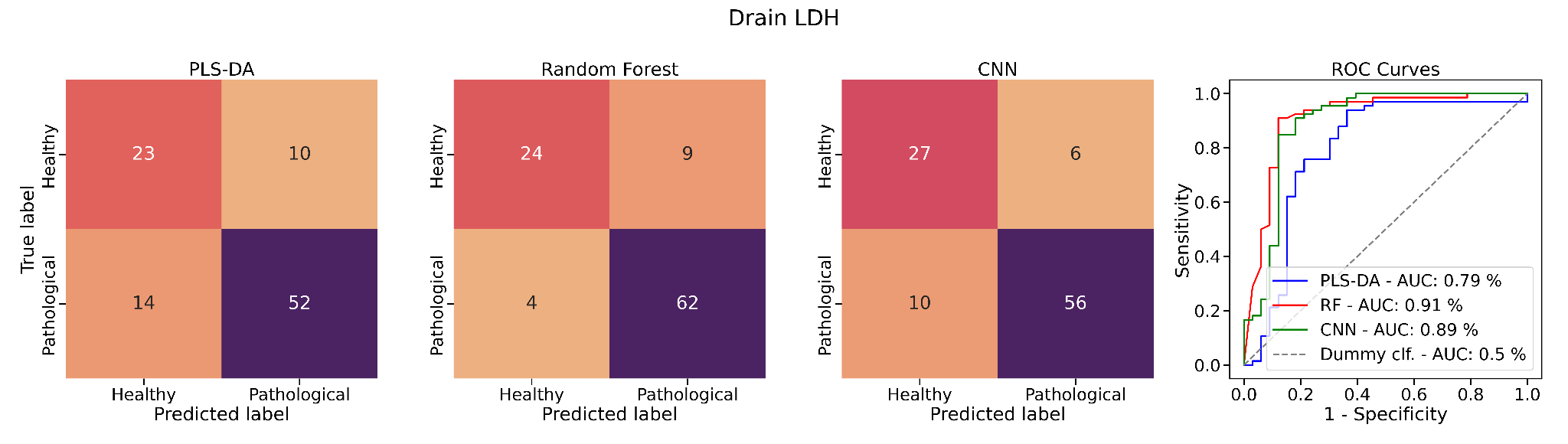


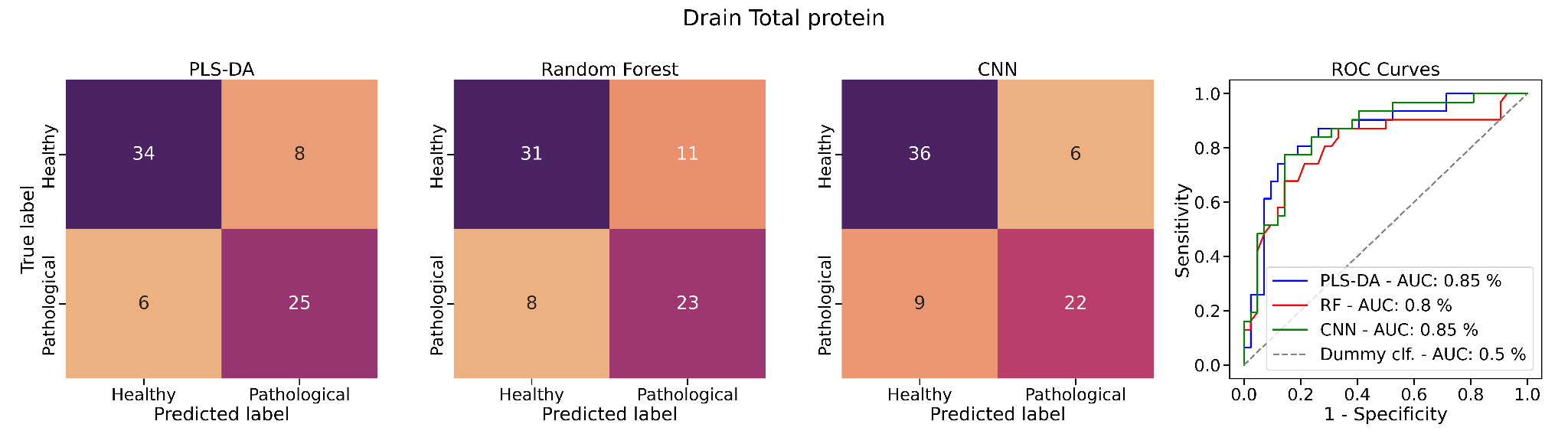


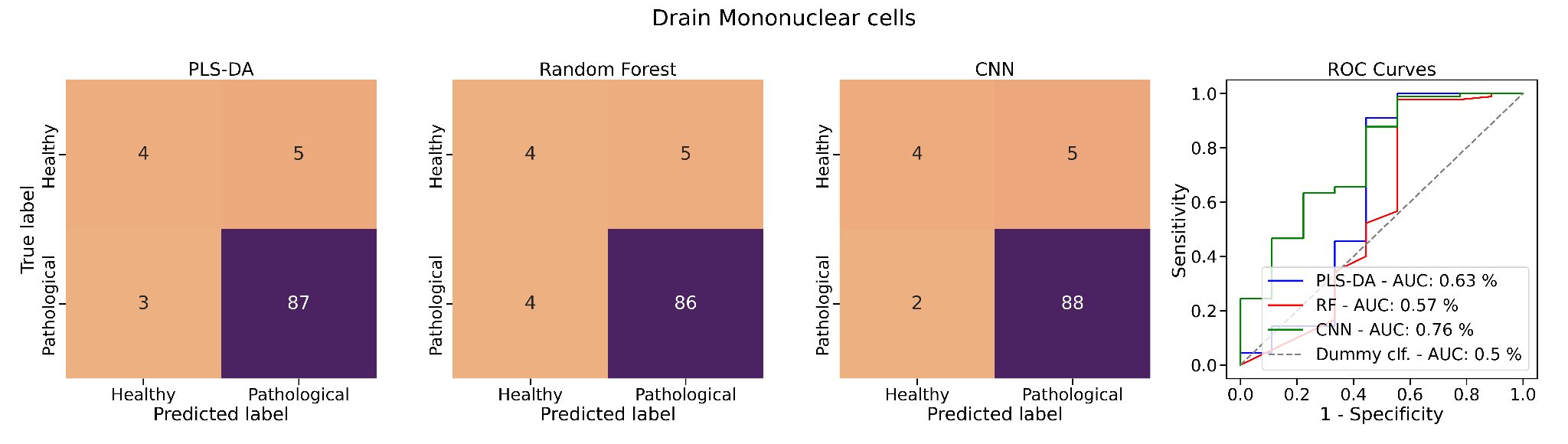


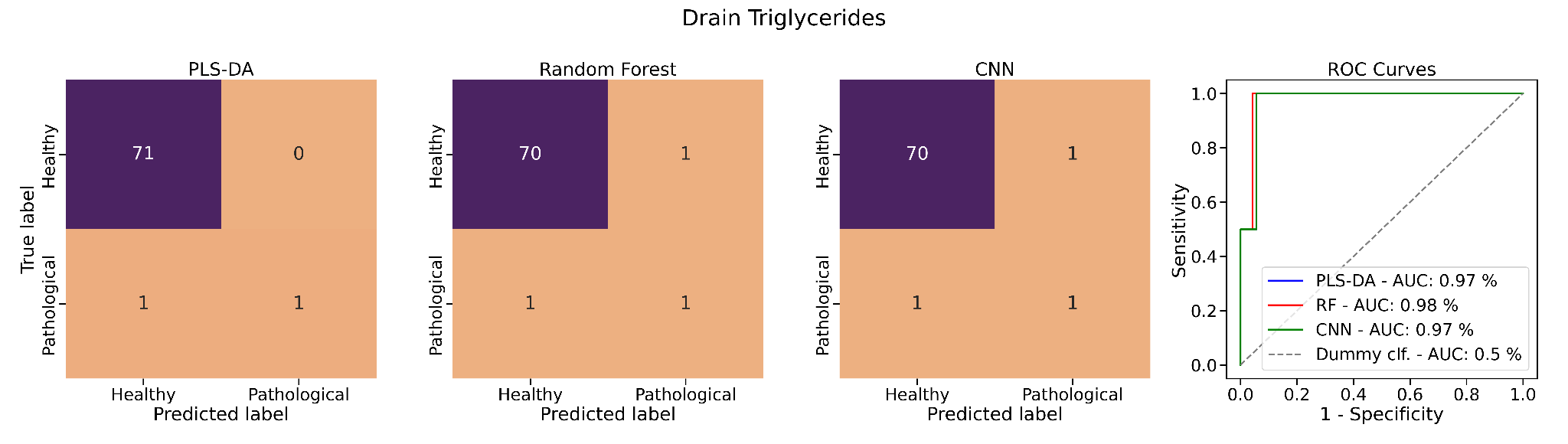


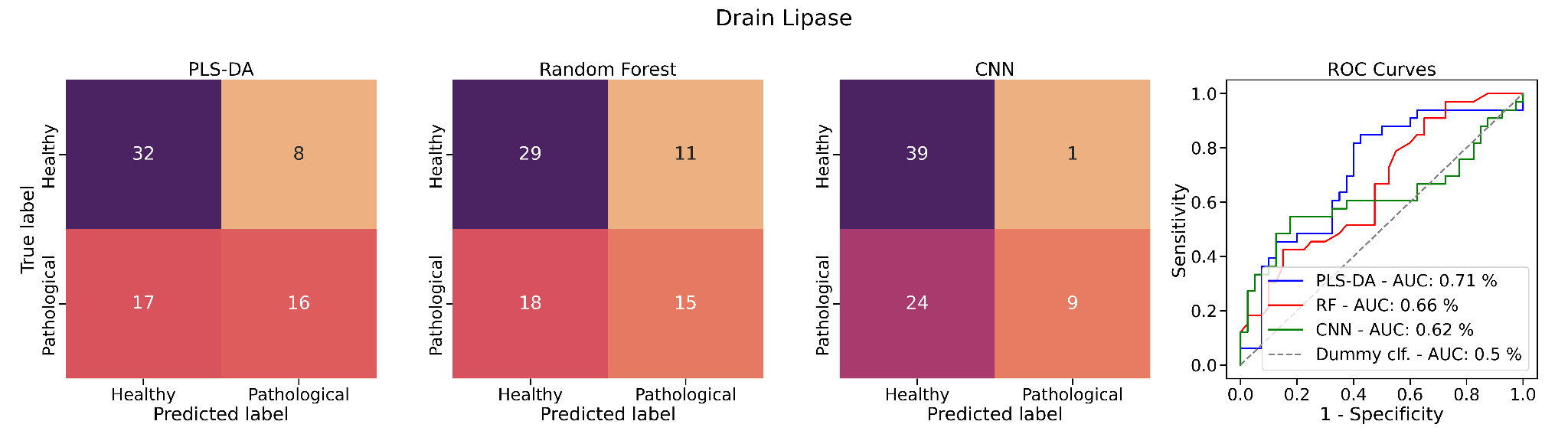


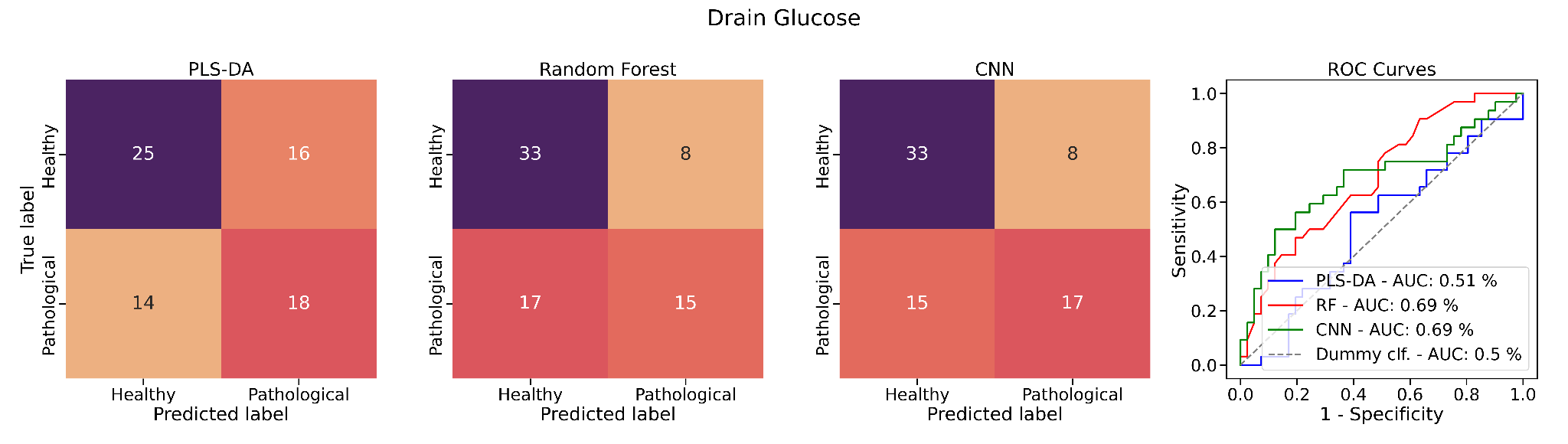


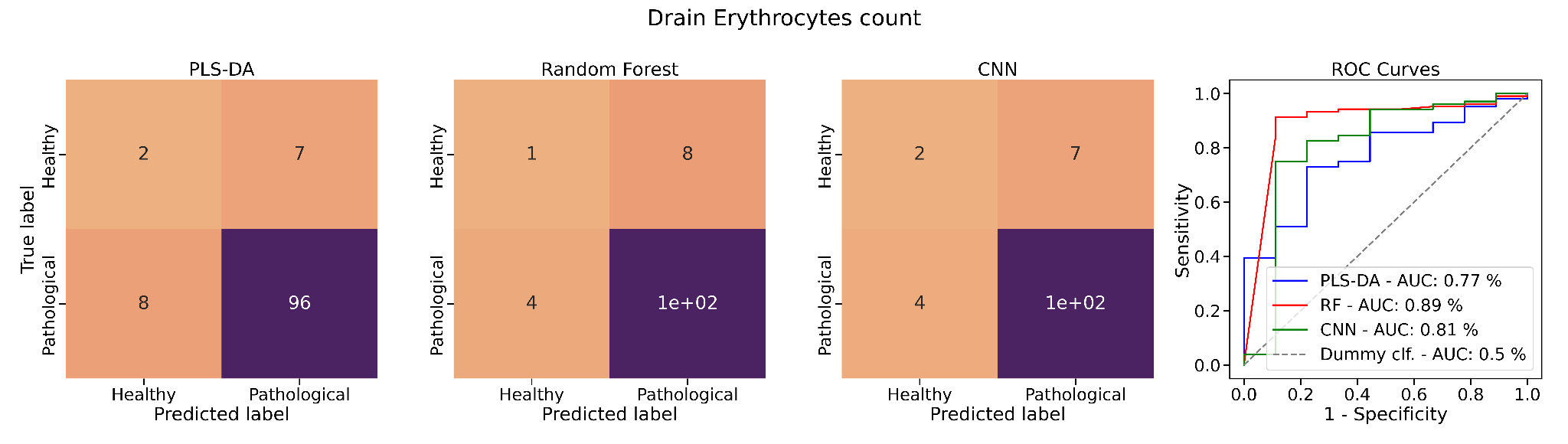


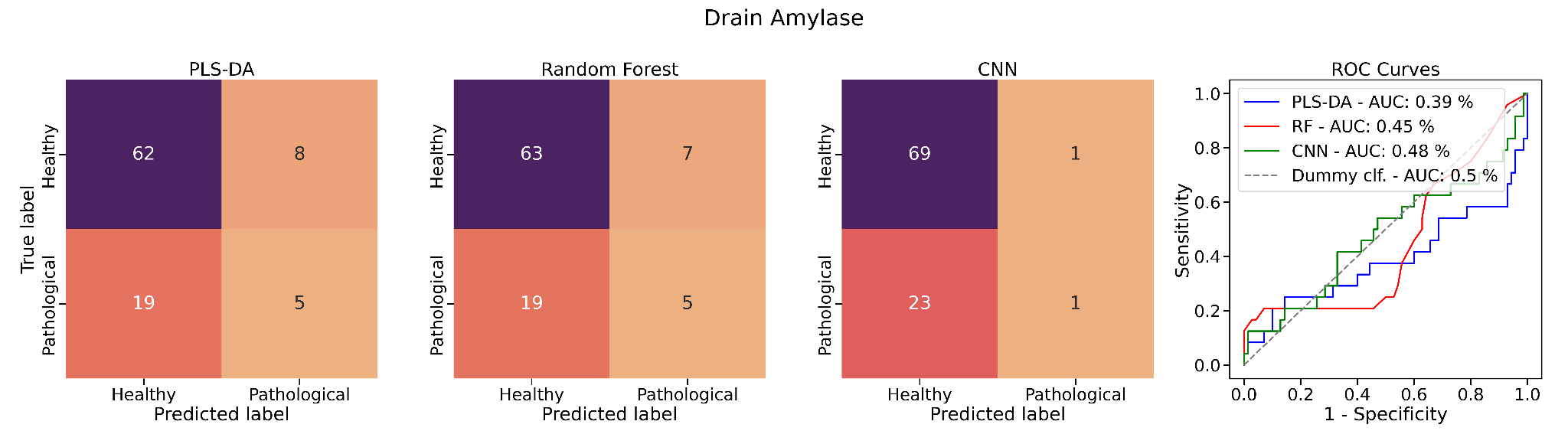


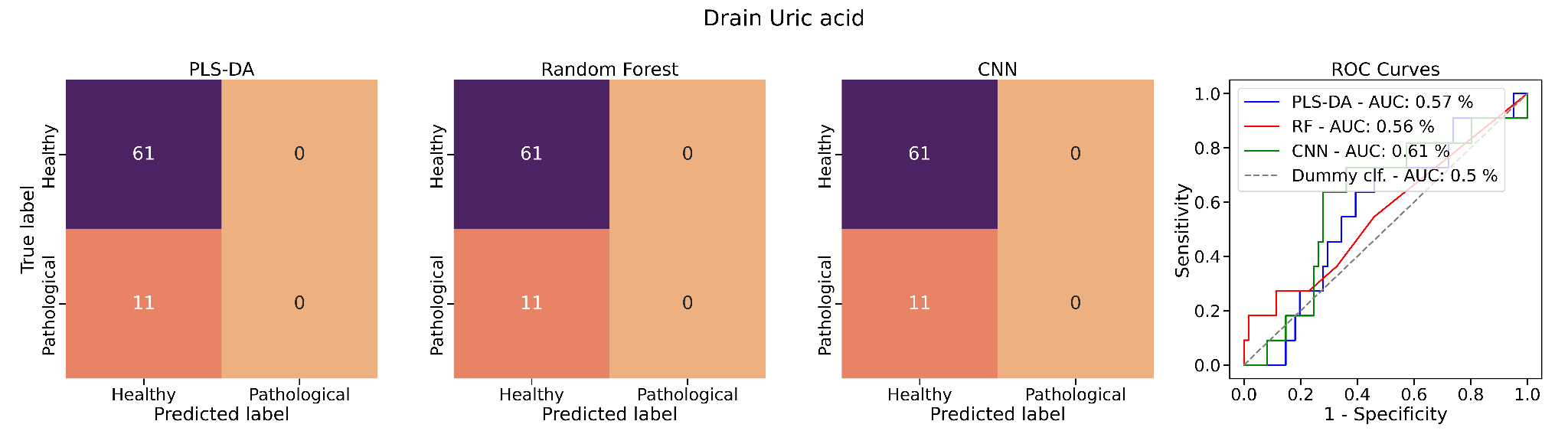


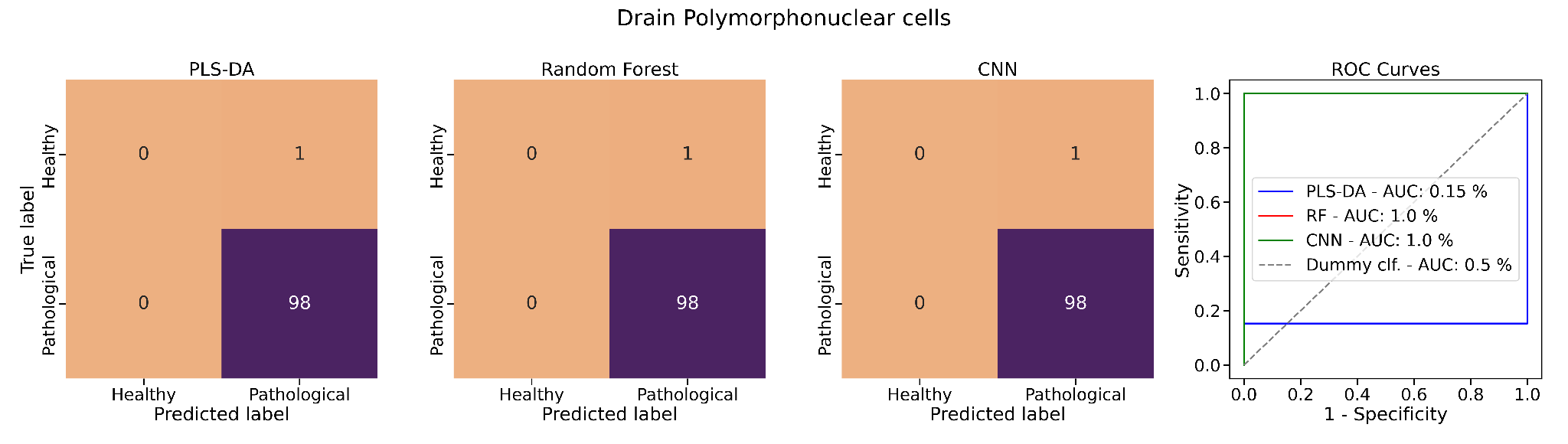


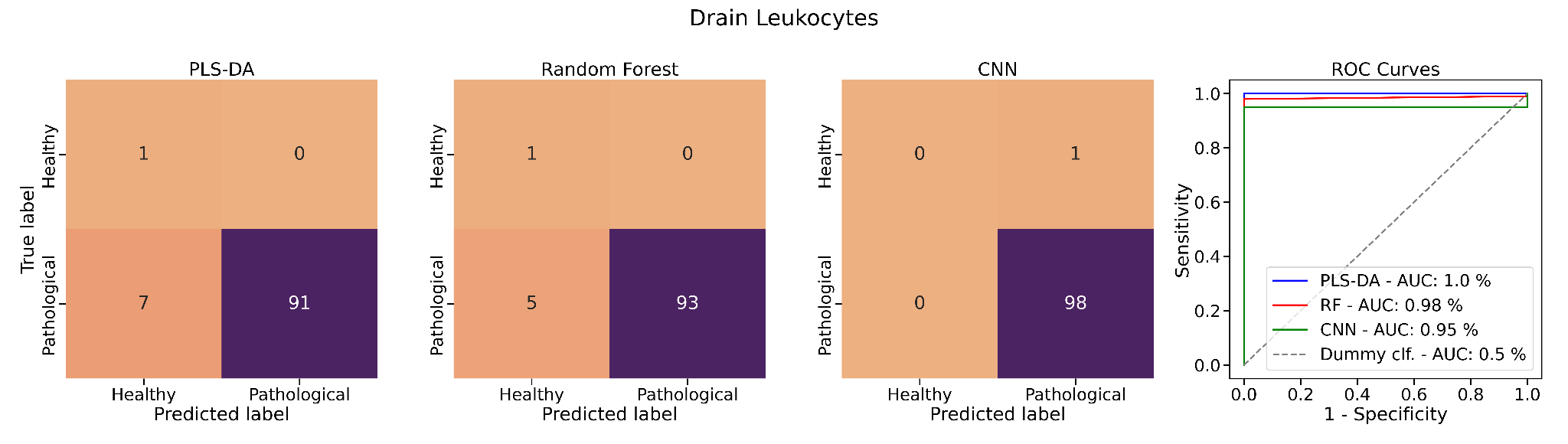


**Figure S2.** Confusion matrices and ROC curves of drainage biomarkers.

## **Figure S3.** Confusion matrices and ROC curves of urine biomarkers models.

####
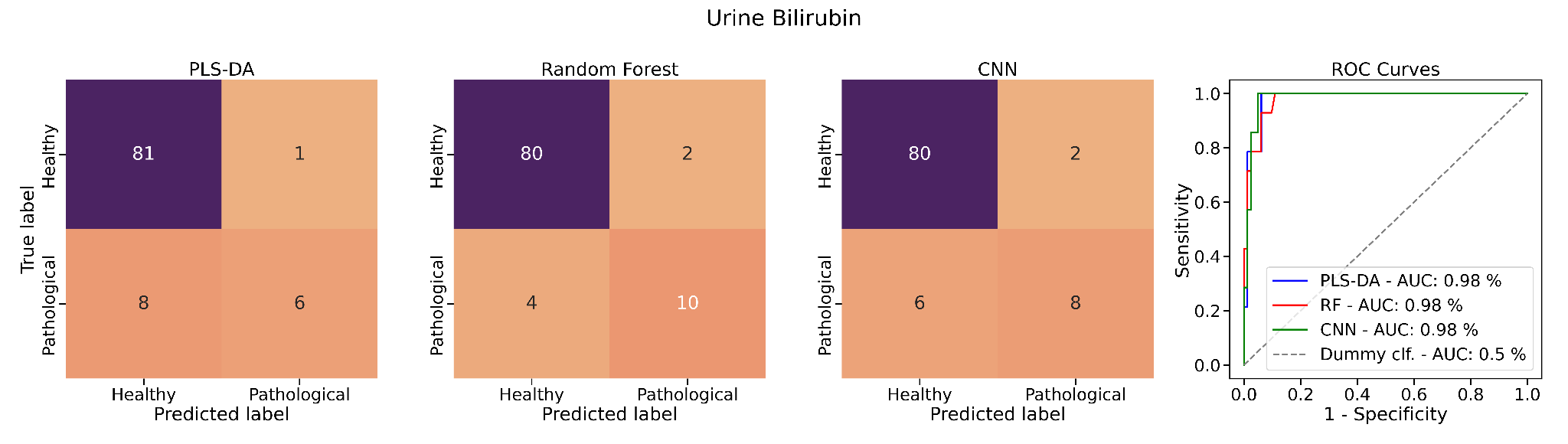

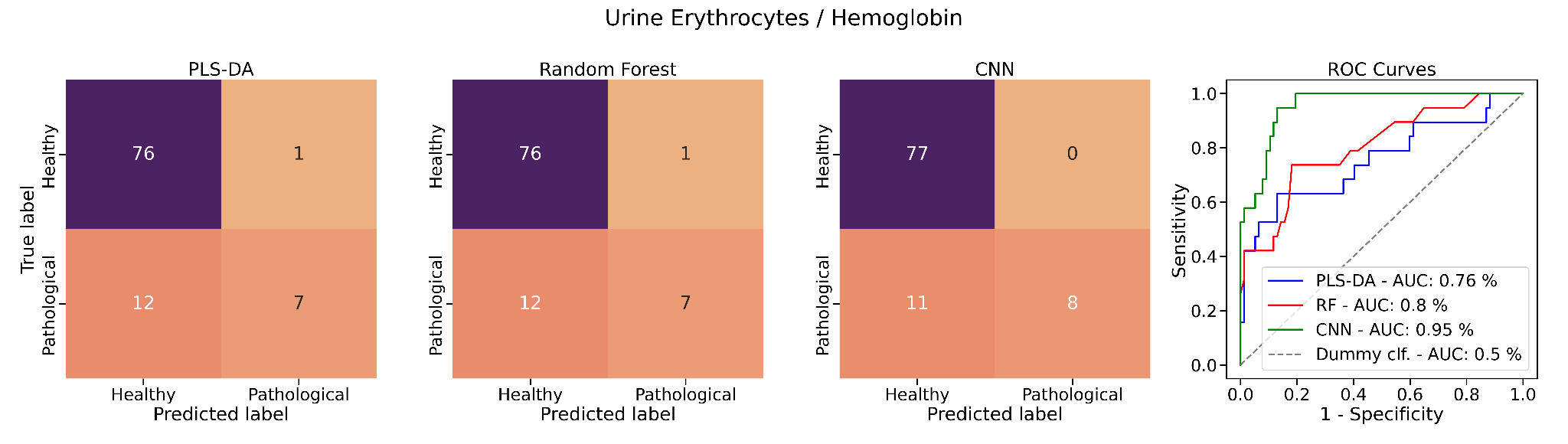


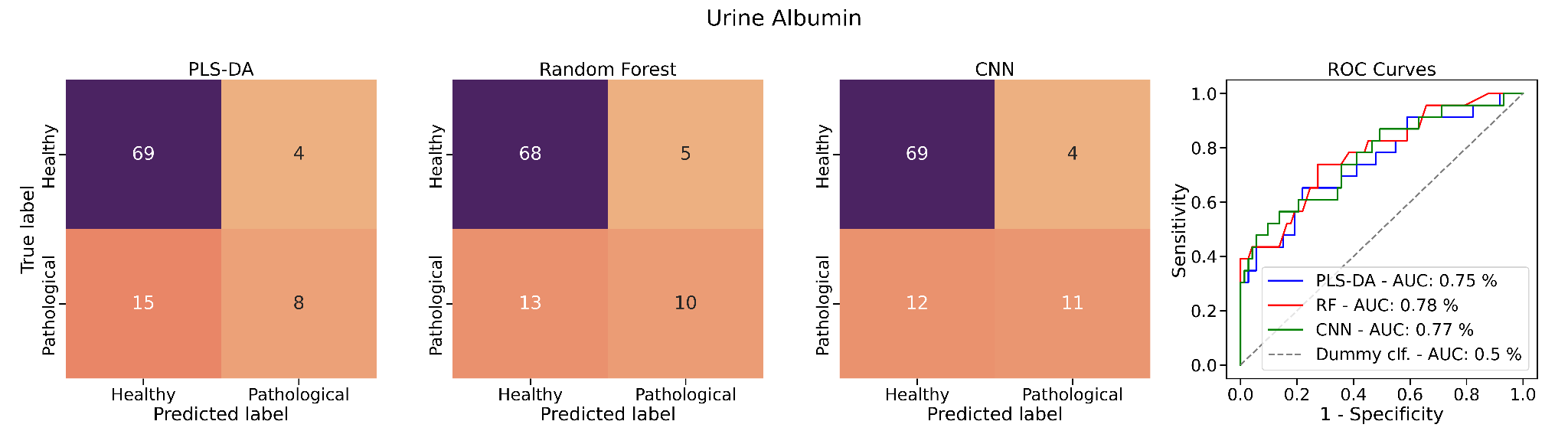


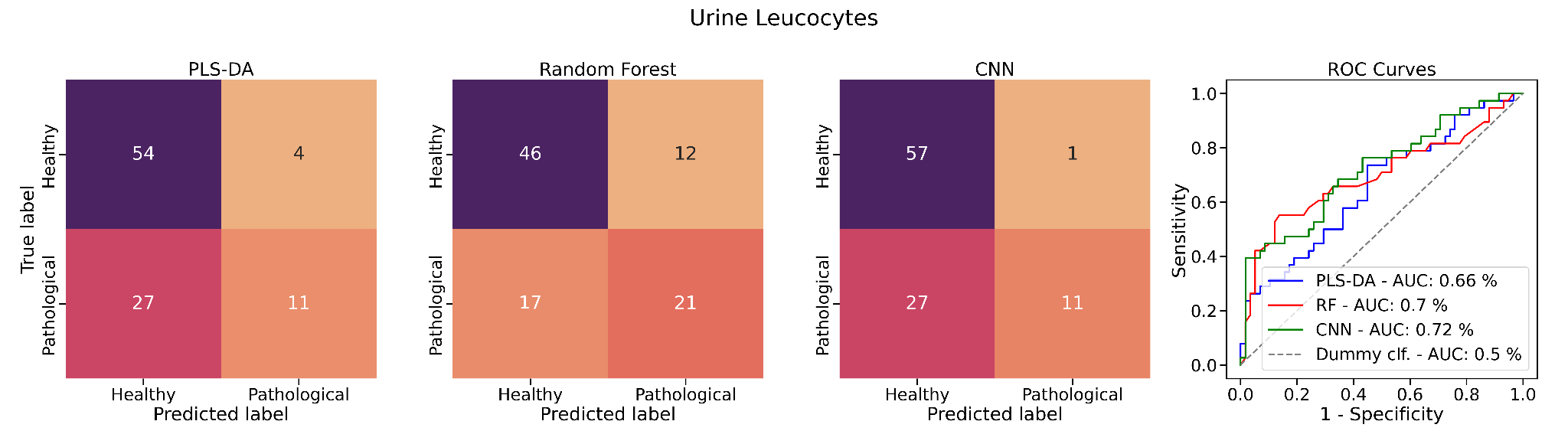


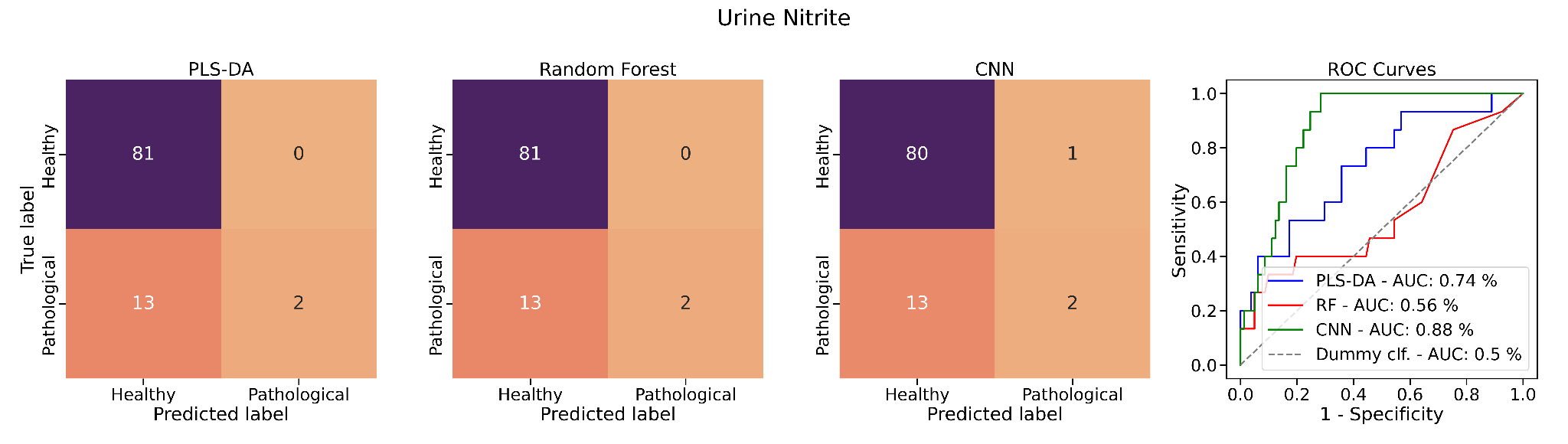


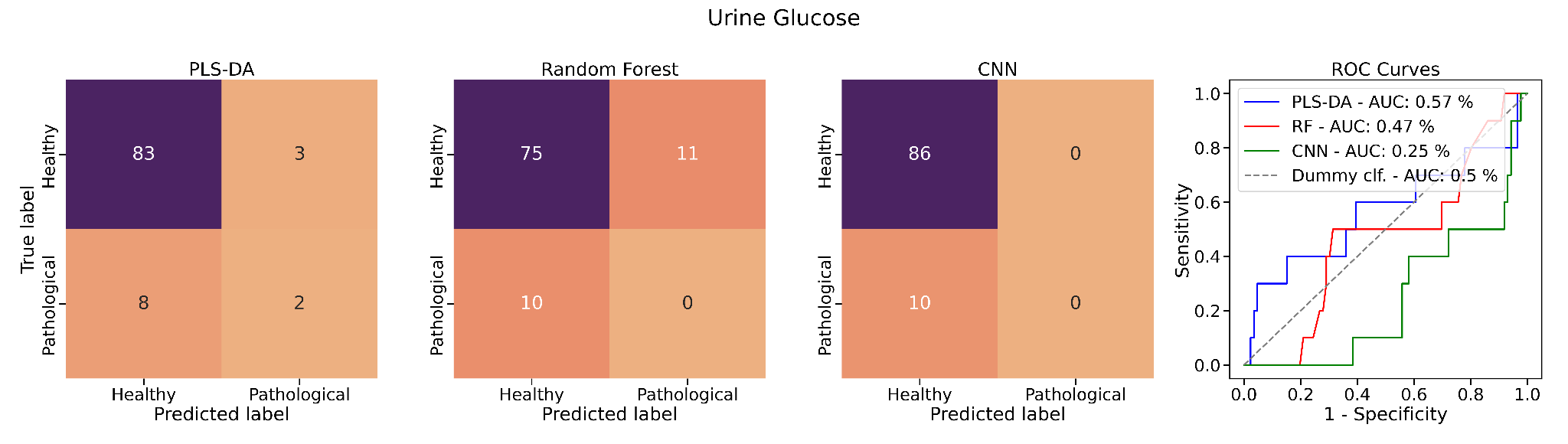


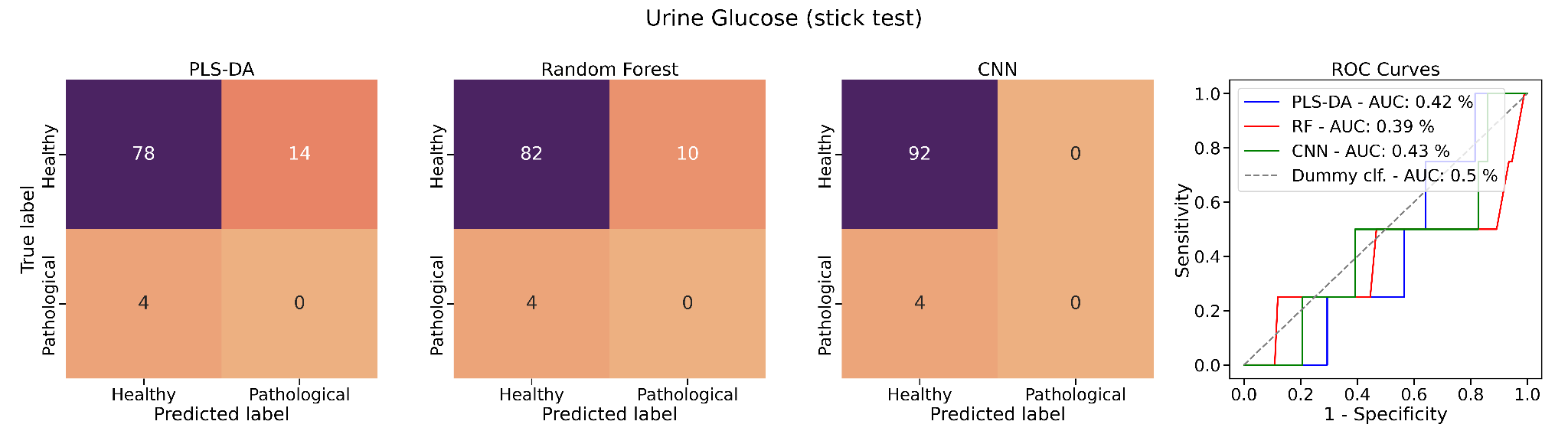


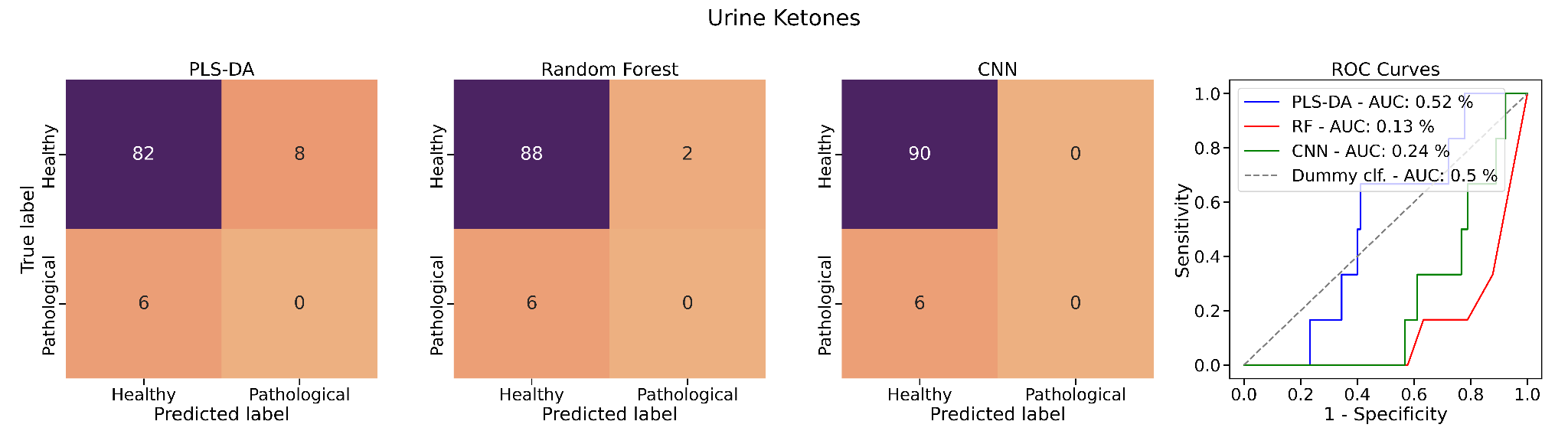


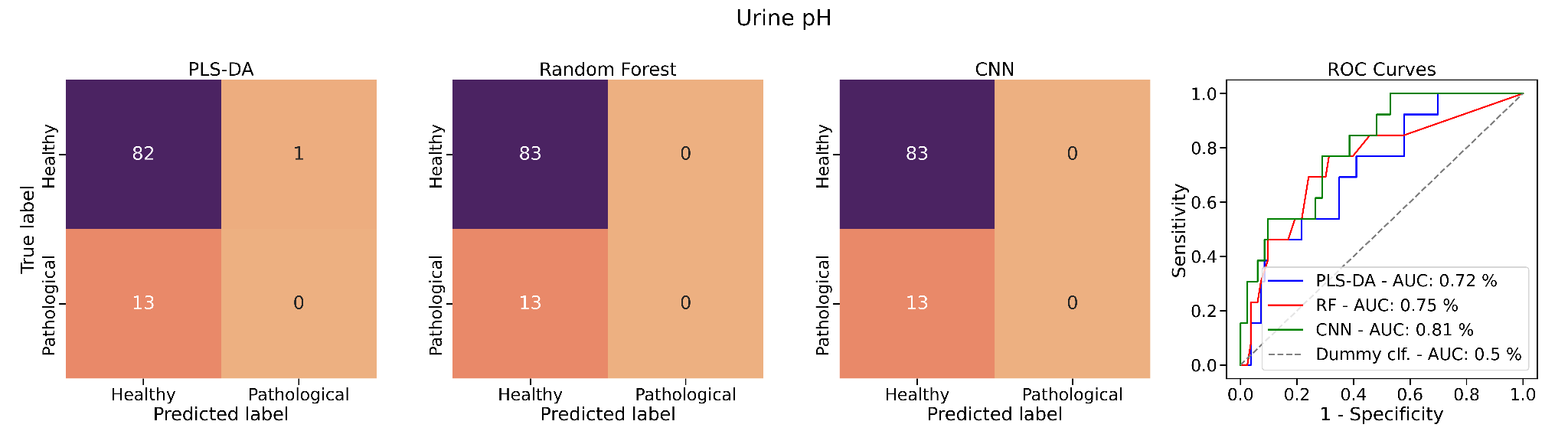


**Figure S3.** Confusion matrices and ROC curves of urine biomarkers.
